# Identification of patients with favorable prognosis after resection in intermediate-stage-hepatocellular carcinoma

**DOI:** 10.1097/JS9.0000000000000941

**Published:** 2023-11-24

**Authors:** Han Ah Lee, Minjong Lee, Jeong-Ju Yoo, Ho Soo Chun, Yewan Park, Hwi Young Kim, Tae Hun Kim, Yeon Seok Seo, Dong Hyun Sinn

**Affiliations:** a Department of Internal Medicine, Ewha Womans University College of Medicine, Seoul, Korea; b Department of Internal Medicine, Ewha Womans University Medical Center; c The Korean Liver Cancer Association; d Department of Medicine, Samsung Medical Center, Sungkyunkwan University School of Medicine; e Department of Internal Medicine, Kyung Hee University Hospital; f Department of Internal Medicine, Korea University College of Medicine, Seoul; g Department of Internal Medicine, Soonchunhyang University Bucheon Hospital, Bucheon, Korea

**Keywords:** hepatocellular carcinoma, intermediate-stage, resection, trans-arterial chemoembolization

## Abstract

**Backgrounds:**

It is unclear which patients benefit from resection in intermediate-stage-hepatocellular carcinoma (HCC). The authors aimed to identify high-risk patients for early recurrence among patients with resectable intermediate-stage HCC.

**Methods:**

This multicenter retrospective study included patients who underwent resection or trans-arterial chemoembolization (TACE) for intermediate-stage HCC (2008–2019). Multivariable Cox proportional analysis was performed to identify high-risk patients when treated with resection. A prediction score for 2-year recurrence-free survival (RFS) was developed using the training cohort and validated. The 2-year RFS in each risk group was compared with that in TACE group, after propensity score matching (PSM).

**Results:**

A total of 1686 patients were included (480 and 1206 patients in the resection and TACE groups). During a median follow-up of 31.4 months, the 2-year RFS was significantly higher in the resection (47.7%) than in the TACE group (19.8%) [adjusted hazard ratio (aHR)=1.471, 95% CI: 1.199–1.803, *P*<0.001). On multivariate analysis, alpha-fetoprotein ≥5.0 ng/ml (aHR=0.202), ALBI grade ≥2 (aHR=0.709), tumor number ≥3 (aHR=0.404), and maximal tumor size ≥5 cm (aHR=0.323) were significantly associated with the lower risk of 2-year RFS in the resection group. The newly developed Surgery Risk score in BCLC-B (SR-B score) with four significant risk factors showed an area under the curve of 0.801 for the 2-year RFS and was validated. Based on the SR-B score, low-risk patients had a significantly higher 2-year RFS (training: aHR=5.834; validation: aHR=5.675) than high-risk patients (all *P*<0.001) did. In a PSM cohort, a low-risk resection group had a significantly higher (aHR=3.891); a high-risk resection group had a comparable 2-year RFS to those treated with TACE (aHR=0.816).

**Conclusions:**

Resection may be beneficial for resectable intermediate-stage HCC based on the SR-B score.

## Introduction

Highlights

- This multicenter retrospective study involving 1686 patients with resectable intermediate-stage-hepatocellular carcinoma (HCC) found that resection resulted in significantly higher 2-year recurrence-free survival (RFS) compared to trans-arterial chemoembolization (TACE).
- In the resection group, alpha-fetoprotein ≥5.0 ng/ml, ALBI grade ≥2, tumor number ≥3, and maximal tumor size ≥5 cm were significant risk factors for 2-year RFS.
- A newly developed prediction score, the Surgery Risk score in BCLC-B (SR-B score) had a very good predictive accuracy for 2-year RFS after the resection in intermediate-stage HCC, suggesting the potential benefits of resection in resectable intermediate-stage HCC.

Approximately 30% of patients with hepatocellular carcinoma (HCC) are diagnosed with intermediate-stage disease, which is defined as multifocal HCC [exceeding Barcelona Clinic Liver Cancer (BCLC) stage A] with preserved liver function and no cancer-related symptoms, vascular invasion, or extrahepatic spread^1,2^. In intermediate-stage HCC, trans-arterial chemoembolization (TACE) is the standard of care based on the survival benefit compared to best supportive care in previous studies^3^. However, intermediate-stage HCC encompasses the largest subgroup of patients and includes a substantially heterogeneous patient population^4^. TACE is not optimal for all patients with intermediate-stage HCC, considering the individualized patient profiles^5^.

Although recent studies suggested that patients with HCC who show TACE refractoriness can benefit from systemic therapy^6^, studies on the therapeutic role of resection and determining which patients will benefit from it are lacking. Patients with well-defined HCC could be candidates for liver transplantation (LT) if they meet the extended LT criteria. However, because of the limited access to LT, resection could be the optimal treatment option in such patients^7^. Resection could prolong survival in patients with resectable intermediate-stage HCC compared to TACE^8^. Although there were two small studies evaluating the characteristics of patients who could benefit from resection over TACE^9,10^, prognostic scoring system for patients with intermediate-stage HCC who are treated with resection in a large scale is still lacking.

We aimed to identify high-risk patients with intermediate-stage HCC who underwent resection and compare their outcomes with those who underwent TACE.

## Methods

### Study population

Between 2008 and 2019, patients newly diagnosed with intermediate-stage HCC who underwent surgery (with histologically confirmed complete removal of tumor tissue) or TACE [with achieved complete response (CR)] as their first-line of treatment were recruited from the Korean Primary Liver Cancer Registry (KPLCR)–the random and representative sample of patients who are newly diagnosed with primary liver cancer in Korea^11,12^, Samsung Medical Center, Soonchunhyang University Bucheon Hospital, Ewha Womans University Seoul Hospital, Ewha Womans University Mokdong Hospital, Korea University Anam Hospital, and Korea University Ansan Hospital.

The exclusion criteria were as follows: (a) age less than 18 years, (b) not belonged to intermediate-stage (including infiltrative type of HCC), (c) no preserved liver function (Child–Pugh score >7), (d) insufficient follow-up period (<6 months), (e) insufficient clinical or laboratory information, (f) history of HCC or organ transplantation, and (g) prior HCC treatment (Supplementary Fig. 1, Supplemental Digital Content 1, http://links.lww.com/JS9/B388). The work has been reported in line with the strengthening the Reporting of cohort, cross-sectional, and case–control studies in Surgery (STROCSS) criteria (Supplemental Digital Content 2, http://links.lww.com/JS9/B389)^13^.

### Definition of HCC and intermediate-stage

HCC was diagnosed based on histological or dynamic computed tomography (CT) and/or magnetic resonance imaging (MRI) findings: nodules greater than 1 cm with arterial hypervascularity and portal/delayed phase washout. In patients who underwent resection, HCC diagnosis was confirmed by histopathological examination of the surgical samples.

Intermediate-stage HCC or BCLC stage B was defined as multifocal HCC exceeding BCLC stage A, with preserved liver function and no cancer-related symptoms (Eastern Cooperative Oncology Group-performance status 0), vascular invasion, infiltrative type of HCC, or extrahepatic spread^3^.

### Data collection

Baseline characteristics of each patient including liver biochemical test and serum alpha-fetoprotein (AFP), and tumor characteristics including numbers and maximal diameter of tumor based on imaging data were collected. Because we aimed to stratify prognosis of patients before the first treatment of resection or TACE, we collected the data within 1 month before treatment. If the pathological data will be used to determine prognosis, it can be difficult to predict prognosis before the treatment of resection or TACE.

### Clinical outcomes and follow-up

The primary outcome was 2-year recurrence-free survival (RFS). The secondary outcome was 5-year overall survival (OS). The index date was the date when HCC was first detected. The treatment approach was determined by each clinician, taking into consideration HCC guidelines, tumor characteristics, liver function, and technical feasibility. When necessary, treatment decisions were made in consultation with a multidisciplinary team. The main selection criteria for resection were the patient’s ability to endure surgical intervention [preserved general condition, preserved liver function, and Child–Pugh class A or B (≤7)] and the anatomical resectability of the tumor (size, number, and location of the lesions, noninfiltrative HCC, and no macrovascular involvement) as assessed on multiphasic CT or MRI^14^.

Treatment response was evaluated 1 month after the treatment. CR was defined as the disappearance of any intratumoral arterial enhancement in the liver on multiphasic CT or MRI^15^. The patients were followed-up every 3 months for 2 years and every 6 months thereafter, where the patients were clinically and radiologically (multiphasic CT or MRI) examined and biochemically assessed using liver function tests and serum AFP levels. Intrahepatic HCC recurrence was defined by the same criteria applied for initial HCC diagnosis. Extrahepatic recurrence was evaluated by CT or bone scan performed at the discretion of the clinician.

### Procedure

We used data from six university-affiliated hospitals and the Korean Primary Liver Cancer Registry (KPLCR) from 190 hospitals in Korea. Three surgeons in each hospital performed resection, under a low central venous pressure of less than 5 mmHg. Anatomic partial hepatectomy was performed in a standardized manner. If patients had poor liver function, nonanatomic partial hepatectomy was performed. All resections were performed by ligating the feeding vessels and securing histologically confirmed tumor-free margins of at least 2 cm, whenever possible. The surgical extent was determined considering close major intrahepatic vessels and future remnant liver volume. Conventional TACE was performed by specialists with greater than 10 years of experience with the procedure. Under the guidance of digital subtraction angiography, a catheter was inserted into the hepatic artery and a super-selective microcatheter was inserted into the tumor-feeding arteries. Chemotherapeutic agents were manually emulsified with iodized oil (Lipiodol Ultra Fluide; Guerbet). The lipiodol emulsion was injected until the tumor-feeding arteries were saturated. Absorbable gelatin sponge particles were used to embolize the feeding arteries completely.

### Identification of high-risk patients with intermediate-stage HCC in the resection group

To identify high-risk patients with intermediate-stage HCC who underwent resection, a multivariable analysis for the 2-year RFS was performed in the training cohort, which consisted of patients recruited from the KPLCR. A score to identify high-risk patients in the resection group was constructed based on potential covariates from the multivariable and binary regression analysis of the 2-year RFS in the training cohort. The sensitivity and specificity at the cutoff values were calculated using the Youden index (sensitivity + specificity – 1).

The discriminatory performance of the screening model for high-risk patients in the resection group was assessed using the area under the curve (AUC) in the training cohort. Thereafter, it was validated using a validation cohort, which consisted of patients recruited from the six academic teaching hospitals. The AUC values of various prediction models were compared using the Delong test^16^. Details of the hepatoma arterial embolization prognostic (HAP) score^17^, modified HAP-II (mHAP-II) score^18^, up-to-seven criteria^19^, six-and-twelve score^20^, four-and-seven criteria^21^, BCLC-B subclassification^4^, preoperative Early Recurrence After Surgery for Liver tumor (ERASL-pre) model^22^, Kinki criteria^23^, Albumin-platelet index^9^, and Multiplication of tumor maximum diameter and number^10^, are summarized in Supplementary Table 1 (Supplemental Digital Content 1, http://links.lww.com/JS9/B388).

Score performance was represented graphically using calibration plots, which compared the score and actual probabilities of a composite parameter in the training and validation sets. The Hosmer–Lemeshow goodness-of-fit test was used to assess calibration of the high-risk screening model for the resection group. In addition, the performance was verified through internal validation using the bootstrap 1000 times resampling method.

### Statistical analyses

To compare the differences in the baseline characteristics between the resection and TACE groups, continuous variables were analyzed using Student’s *t*-test or Mann–Whitney *U* test. The categorical variables were analyzed using the chi-squared or Fisher’s exact test when appropriate. Continuous variables are summarized as mean±SD, and categorical variables are presented as frequencies and percentages.

To minimize potential bias according to the different baseline characteristics between the resection and TACE groups, propensity score (PS) matching was performed by fitting a logistic regression model that included the following variables: age, sex, diabetes, etiology of liver disease, levels of serum albumin, total bilirubin, international normalized ratio (INR), alanine aminotransferase (ALT), and AFP, platelet count, albumin-bilirubin (ALBI) grade, MELD score, Child–Pugh class, and tumor number (≥3) and maximal tumor size (≥5 cm). A 1:1 ratio PS-matching was performed using the nearest-neighbor method on the logit of PS. The caliper width was 0.02 times the SD of the logit of PS.

RFS and OS were calculated using the Kaplan–Meier method and compared using the log-rank test. The data of patients who experienced recurrence, died, or received LT during the follow-up period were censored. Independent predictors for 2-year RFS and 5-year OS were evaluated using Cox proportional hazard regression analysis. To generate the risk prediction model, we attempted to identify significant risk factors associated with 2-year RFS and constructed the prediction model in the training cohort using selected risk factors. The multivariate Cox proportional hazards model was used to calculate the β regression coefficient, *P*-value, adjusted hazard ratio (HR) and its 95% CI for each of the selected risk predictors. Variables with *P*<0.05 were incorporated into the risk prediction model. All statistical analyses were performed using SPSS software (version 21.0; SPSS, Inc.). *P*<0.05 was considered statistically significant.

## Results

### Patient characteristics

After excluding subjects who met our exclusion criteria, 1686 patients were selected for statistical analysis [480 (28.5%) and 1206 (71.5%) in the resection and TACE groups, respectively; Supplementary Fig. 1, Supplemental Digital Content 1, http://links.lww.com/JS9/B388]. Among patients in the resection group, five patients had adjuvant therapy after resection (three patients received chemotherapy and two patients received radiotherapy). Among patients who underwent TACE, only four patients (1.3%) had combined RFA. The baseline characteristics of the study population are presented in Table 1. The mean MELD score was significantly lower in the resection group than in the TACE group (7.00 vs. 8.09, *P*<0.001). The proportion of patients with ALBI grade 1 was significantly higher in the resection group (*n*=345, 71.9%) than in the TACE group (*n*=586, 48.6%). The proportion of patients with greater than or equal to 3 tumors was significantly lower in the resection group (*n*=106, 22.1%) than in the TACE group (*n*=771, 63.9%; *P*<0.001). The proportion of patients with a maximal tumor size of greater than or equal to 5 cm was significantly larger in the resection group (*n*=217, 45.2%) than in the TACE group (*n*=454, 37.6%; *P*=0.001) (Table 1). The clinical characteristics of the patients balanced by PS-matching (419 vs. 419 patients in the resection and TACE groups, respectively) are presented in Table 1.

**Table 1:** Baseline characteristics of all patients and patients balanced by propensity score matching.

	All patients				Propensity score-matched patients				
	All patient (*n*=1686)	Resection group (*n*=480, 28.5%)	TACE group (*n*=1206, 71.5%)	*P*	All patients	Resection group (*n*=838)	TACE group (*n*=419, 50.0%)	*P*	Standardized difference
Age	60.0 (53.0–68.0)	59.0 (53.0–68.0)	60.0 (53.0–68.0)	0.786	60.0 (53.0–68.0)	60.0 (53.0–69.0)	60.0 (52.0–68.0)	0.314	0.069
Male, *n* (%)	1415 (83.9)	401 (83.5)	1,014 (84.1)	0.903	712 (85.0)	355 (84.7)	357 (85.2)	0.923	0.013
Diabetes, *n* (%)	444 (26.3)	108 (22.5)	336 (27.9)	0.052	215 (25.7)	100 (23.9)	115 (27.4)	0.268	−0.086
Viral etiology, *n* (%)	1324 (78.5)	351 (73.1)	973 (80.7)	<0.001	632 (75.4)	106 (25.3)	100 (23.9)	0.688	0.032
Serum albumin, g/dl	4.0 (3.6–4.3)	4.2 (3.8–4.4)	3.9 (3.5–4.2)	<0.001	4.1 (3.8–4.4)	4.1 (3.8–4.4)	4.1 (3.8–4.4)	0.771	−0.020
Total bilirubin, mg/dl	0.8 (0.6–1.1)	0.8 (0.5–0.9)	0.7 (0.6–1.2)	<0.001	0.7 (0.5–1.0)	0.7 (0.5–1.0)	0.7 (0.5–1.0)	0.786	−0.018
INR	1.08 (1.01–1.16)	1.10 (1.0–1.1)	1.03 (1.0–1.2)	<0.001	1.06 (1.00–1.12)	1.04 (1.00–1.11)	1.07 (1.01–1.13)	0.771	−0.018
Alanine aminotransferase, IU/l	39.0 (25.0–59.0)	36.0 (24.0–54.0)	40.0 (26.0–61.0)	0.909	36.0 (24.0–57.0)	36.0 (24.0–54.0)	38.0 (25.0–59.0)	0.656	−0.023
Platelet count, ×10^9^/l	149.0 (106.0–199.0)	185.5 (143.8–226.0)	134.0 (94.0–181.3)	<0.001	170.5 (130.0–216.0)	175.0 (132.0–217.0)	166.0 (124.0–211.0)	0.240	0.087
Alpha-fetoprotein, ng/ml	41.1 (8.4–521.1)	17.2 (4.7–351.0)	50.8 (10.7–609.1)	0.623	27.6 (6.2–495.5)	15.8 (4.5–285.8)	37.0 (8.3–804.9)	0.286	−0.102
ALBI grade, *n* (%)				<0.001				0.052	−0.023
1	931 (55.2)	345 (71.9)	586 (48.6)		560 (66.8)	286 (68.3)	274 (65.4)		
2	721 (42.8)	126 (26.3)	595 (49.3)		267 (31.9)	124 (29.6)	143 (34.1)		
3	34 (2.0)	9 (1.9)	25 (2.1)		11 (1.3)	9 (2.1)	2 (0.5)		
MELD score	7.70 (6.96–9.05)	7.00 (6.48–8.00)	8.09 (7.12–9.48)	<0.001	7.29 (6.67–8.19)	7.00 (6.53–8.00)	7.50 (6.86–8.47)	0.786	−0.017
Child–Pugh class, *n* (%)				<0.001				1.000	0.012
A	1,538 (91.2)	457 (95.2)	1,081 (89.6)		801 (95.6)	400 (95.5)	401 (95.7)		
B	148 (8.8)	23 (4.8)	125 (10.4)		37 (4.4)	19 (4.5)	18 (4.3)		
Tumor number ≥3, *n* (%)	877 (52.0)	106 (22.1)	771 (63.9)	<0.001	234 (27.9)	112 (26.7)	122 (29.1)	0.488	−0.057
Maximal tumor size ≥5 cm, *n* (%)	671 (39.8)	217 (45.2)	454 (37.6)	0.001	382 (45.6)	193 (46.1)	189 (45.1)	0.835	0.019

Variables are expressed as median (interquartile range) or *n* (%). TACE, trans-arterial chemoembolization; INR, international normalized ratio; ALBI, albumin-bilirubin; MELD, model for end-stage liver disease.

### Favorable RFS and OS in the resection group after PS-matching analysis

**Figure 1:**
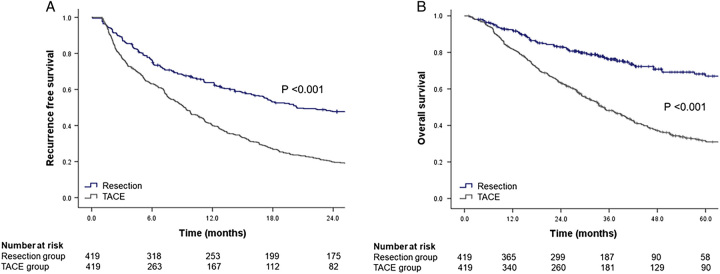
The survival outcomes of resection and TACE groups.The 2-year recurrence-free survival was significantly higher in the resection group than in the TACE group (A), and The 5-year overall survival was significantly higher in the resection group than in the TACE group in propensity score-matched cohorts (B). TACE, trans-arterial chemoembolization.

The median follow-up period in this study was 31.4 months [interquartile range (IQR) 15.8–48.4 months]. In the PS-matched cohort, RFS was significantly higher in the resection group than in the TACE group (*P*<0.001, log-rank test; Fig. 1A). In the resection group, the median RFS was 20.4 months (95% CI: 15.0–25.8 months); the 1-year, 2-year, and 5-year RFS were 63.8, 47.7, and 20.9%, respectively. In the TACE group, the median RFS was 9.2 months (95% CI: 7.9–10.6 months): the 1-year, 2-year, and 5-year RFS were 40.3, 19.8, and 6.4%, respectively. Resection was associated with a significantly higher rate of 2-year RFS [adjusted hazard ratio (aHR)=1.471, 95% CI: 1.199–1.803, *P*<0.001], compared to TACE. The OS was significantly higher in the resection group than in the TACE group (*P*<0.001, by log-rank test; Fig. 1B). In the resection group, the median OS was not reached; the 1-year, 2-year, and 5-year OS rates were 92.5, 83.0, and 67.0%, respectively. In the TACE group, the median OS was 34.6 months (95% CI: 30.0–39.1 months); the 1-year, 2-year, and 5-year OS rates were 81.8, 63.2, and 31.7%, respectively. Resection was significantly associated with a higher 5-year OS (aHR=6.181, 95% CI: 4.670–8.181, *P*<0.001) than TACE was.

### Predictive factors for the 2-year RFS in the resection group

To develop a new prognostic score system for stratifying 2-year RFS, patients in the resection group were divided into training (KPLCR data, 311 patients, 64.8%) and validation (six university-affiliated hospitals, 169 patients, 35.2%) cohorts (Table 2).

**Table 2:** Clinical characteristics of training and validation cohorts of the resection group.

	Training cohort (*n*=311)	Validation cohort (*n*=169)	*P*
Age	59.0 (52.0–68.0)	60.0 (55.0–70.0)	0.119
Male, *n* (%)	258 (83.0)	143 (84.6)	0.640
Diabetes, *n* (%)	71 (22.8)	37 (21.9)	0.815
Viral etiology, *n* (%)	232 (74.6)	119 (70.4)	0.323
Serum albumin, g/dl	4.1 (3.8–4.4)	4.2 (3.8–4.4)	0.186
Total bilirubin, mg/dl	0.7 (0.5–1.0)	0.7 (0.4–0.9)	0.165
INR	1.03 (0.99–1.10)	1.04 (1.00–1.11)	0.879
Alanine aminotransferase, IU/l	37.0 (24.0–56.0)	34.0 (23.0–52.0)	0.861
Platelet count, ×10^9^/l	182.0 (137.0–224.0)	194.0 (151.0–234.5)	0.042
Alpha-fetoprotein, ng/ml	15.6 (4.7–303.8)	21.0 (4.6–495.3)	0.871
ALBI grade, *n* (%)			0.285
1	220 (70.7)	125 (74.0)	
2	83 (26.7)	43 (25.4)	
3	8 (2.6)	1 (0.6)	
MELD score	7.0 (6.4–8.0)	7.0 (6.7–8.0)	0.769
Child–Pugh class, *n* (%)			0.166
A	293 (94.2)	164 (97.0)	
B	18 (5.8)	5 (3.0)	
Tumor number ≥3, *n* (%)	82 (24.8)	34 (18.9)	0.247
Maximal tumor size ≥5 cm, *n* (%)	145 (43.9)	90 (50.0)	0.161

Variables are expressed as median (interquartile range) or *n* (%).

ALBI, albumin-bilirubin; INR, international normalized ratio; MELD, model for end-stage liver disease.

In the multivariable analysis, serum AFP level greater than or equal to 5.0 ng/ml (aHR=0.202, 95% CI: 0.121–0.336, *P*<0.001), ALBI grade greater than or equal to 2 (aHR=0.709, 95% CI: 0.511–0.983, *P*=0.039), tumor numbers greater than or equal to 3 (aHR=0.404, 95% CI: 0.287–0.568, *P*<0.001), and maximal tumor size greater than or equal to 5 cm (aHR=0.323, 95% CI: 0.233–0.448, *P*<0.001) were significant predictors of 2-year RFS (Table 3).

**Table 3:** Predictors for 2-year recurrence-free survival in the training cohort of the resection group.

		Univariate analysis			Multivariable analysis		
Variables	Rating	HR	95% CI	*P*	HR	95% CI	*P*
Age	years	1.006	0.992–1.022	0.398			
Sex	0=women; 1=men	0.981	0.647–1.489	0.929			
Diabetes	0=no; 1=yes	0.892	0.608–1.307	0.557			
Viral etiology	0=other; 1=viral	0.710	0.471–1.069	0.101			
Alanine aminotransferase	IU/l	1.001	0.999–1.004	0.283			
Platelet count	×10^9^/l	1.000	0.997–1.003	0.989			
Alpha-fetoprotein	≥5.0 ng/ml	0.240	0.145–0.398	<0.001	0.202	0.121–0.336	<0.001
ALBI grade	≥2	1.488	1.053–2.101	0.024	0.709	0.511–0.983	0.039
Tumor number	≥3	0.512	0.366–0.716	<0.001	0.404	0.287–0.568	<0.001
Maximal tumor size	≥5 cm	0.372	0.270–0.512	<0.001	0.323	0.233–0.448	<0.001

ALBI, albumin-bilirubin; HR, hazard ratio; MELD, model for end-stage liver disease; TACE, trans-arterial chemoembolization.

Based on the results of the multivariable analysis for 2-year RFS, we established the surgery risk score in BCLC-B (SR-B score) using the following formula: the SR-B score=2.361×AFP (0: <5.0 ng/ml; 1: ≥5.0 ng/ml) + 0.581×ALBI grade (0: Grade 1; 1: Grade 2 or 3) + 1.460×tumor number (0: two; 1: three or more) + 1.624×maximal tumor size (0: <5 cm; 1: ≥5 cm) +1.154. The AUC of the SR-B score for predicting 2-year RFS after resection was 0.801 (95% CI: 0.753–0.849) in the training cohort (Supplementary Figure 2A, Supplemental Digital Content 1, http://links.lww.com/JS9/B388). The optimal cutoff value of the SR-B score was was 3.81 with a sensitivity of 81.0% and a specificity of 78.3%.

### Validation of the SR-B score for 2-year RFS in the resection group

The SR-B score was externally validated in the validation cohort and internally validated in the entire cohort using the bootstrap method. The AUC value of the SR-B score was 0.801 (95% CI: 0.733–0.866, *P*<0.001) in the validation cohort (Supplementary Figure 2B, Supplemental Digital Content 1, http://links.lww.com/JS9/B388) and 0.800 (95% CI: 0.761–0.839, *P*<0.001) in the entire cohort treated with resection (Supplementary Figure 2C, Supplemental Digital Content 1, http://links.lww.com/JS9/B388). The calibration plots showed an overall good agreement between the SR-B score predictions and the observed outcomes in the training and validation cohorts (*P*-value for Hosmer–Lemeshow test=0.105 and 0.702 in the training and validation cohorts, respectively) (Supplementary Figure 3, Supplemental Digital Content 1, http://links.lww.com/JS9/B388).

The diagnostic accuracies of the various prediction models for predicting 2-year RFS were compared in the validation cohort. The AUC of the SR-B score was significantly higher than those of the serum AFP, HAP, mHAP-II, and six-and-twelve scores, up-to-seven criteria, four-and-seven criteria, BCLC-B subclassification, ERASL-pre-score, Kinki criteria, Albumin-platelet index, and Multiplication of tumor maximum diameter and number (all *P*<0.001 by Delong’s test) (Supplementary Table 2, Supplemental Digital Content 1, http://links.lww.com/JS9/B388).

### Risk stratification for the 2-year RFS in the resection group

**Figure 2:**
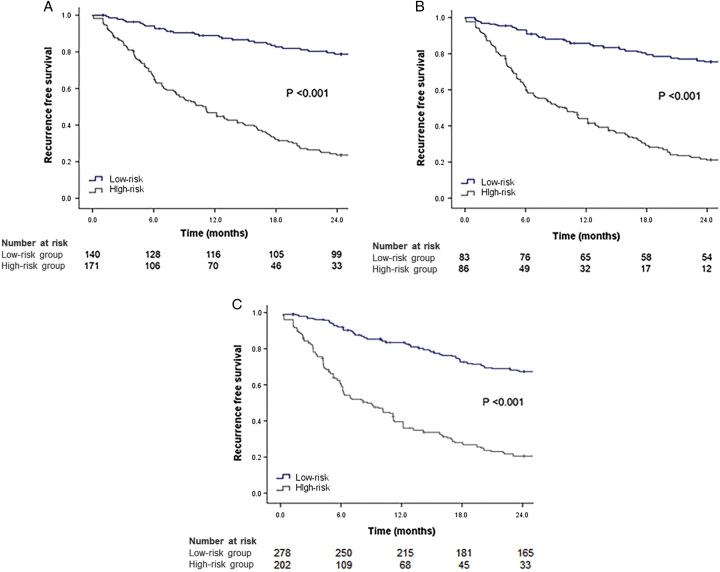
The 2-year recurrence-free survival of patients treated with resection based on the SR-B score. The 2-year recurrence-free survival was significantly higher in the low-risk group than in the high-risk group according to the SR-B score in the training cohort (A), validation cohort (B), and entire cohort (C). SR-B score, surgery risk score in BCLC-B.

According to the SR-B score, patients in the resection group were categorized as low-risk (<4.24, *n*=223, 46.5%) and high-risk (≥4.24, *n*=257, 53.5%) groups (Supplementary Table 3, Supplemental Digital Content 1, http://links.lww.com/JS9/B388). The 2-year RFS was significantly higher in the low-risk group than in the high-risk group (aHR=5.834, 95% CI: 3.900–8.728, *P*<0.001 in the training cohort; aHR=5.675, 95% CI: 3.356–9.595, *P*<0.001 in the validation cohort; aHR=5.852, 95% CI: 4.226–8.103, *P*<0.001 in the entire cohort) (Fig. 2A–C).

### Comparison of the outcomes in patients treated with resection or TACE according to the SR-B scores

**Figure 3:**
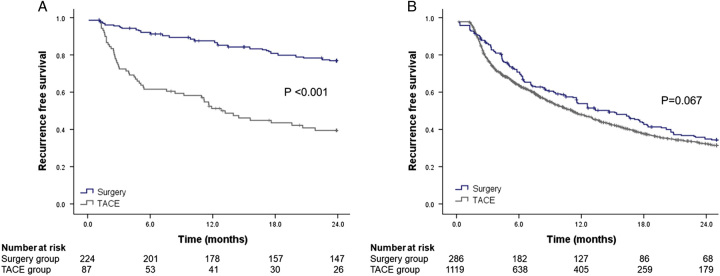
The 2-year recurrence-free survival of resection and TACE groups according to the risk groups classified with the SR-B score. In the low-risk group, the 2-year recurrence-free survival was significantly higher in the resection group than in the TACE group (A). In the high-risk group, the 2-year recurrence-free survival was comparable between the resection and TACE groups (B). SR-B score, surgery risk score in BCLC-B; PS, propensity score; TACE, trans-arterial chemoembolization.

Among entire cohort (*n*=1686), the 2-year RFS was 36.8% in the low-risk group (*n*=311) and 13.3% in the high-risk group (*n*=1375) according to the SR-B scores. In the low-risk group, patients in the resection group had a significantly higher 2-year RFS than in the TACE group (aHR=0.162, 95% CI: 0.087–0.302, *P*<0.001; *P*<0.001 by log-rank test) (Fig. 3A). However, in the high-risk group, the 2-year RFS was comparable between the resection and TACE groups (aHR=0.943, 95% CI: 0.787–1.131, *P*=0.529; *P*=0.067 by log-rank test) (Fig. 3B).

In the PS-matched cohort, according to the SR-B scores, low-risk patients in the resection group (*n*=178) had a significantly higher 2-year RFS than those in the TACE group (*n*=178) (aHR=3.891, 95% CI: 2.633–5.750, *P*<0.001; *P*<0.001 by log-rank test, Supplementary Figure 4A, Supplemental Digital Content 1, http://links.lww.com/JS9/B388). However, high-risk patients in the resection group (*n*=257) had a comparable 2-year RFS to those in the TACE group (*n*=257) (HR=0.816, 95% CI: 0.653–1.019, *P*=0.073; *P*=0.072 by log-rank test, Supplementary Figure 4B, Supplemental Digital Content 1, http://links.lww.com/JS9/B388). In the PS-matched cohort, the 5-year overall survival in the resection group was significantly higher than the TACE group: in the low-risk group (aHR=3.747, 95% CI: 2.319–6.054, *P*<0.001; Supplementary Figure 5A, Supplemental Digital Content 1, http://links.lww.com/JS9/B388) and in the high-risk group (aHR=2.305, 95% CI: 1.780–2.984, *P*<0.001; Supplementary Figure 5B, Supplemental Digital Content 1, http://links.lww.com/JS9/B388) according to the SR-B score.

### Higher 5-year overall survival in the resection group than in the TACE group who has the risk of poor prognosis

**Figure 4:**
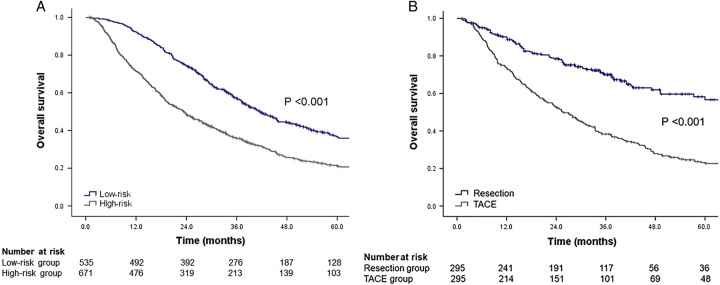
The 5-year overall survival of risk groups based on the HAP score. In the TACE group, the 5-year overall survival was significantly higher in the low-risk group than in the high-risk group according to the HAP score (A). The 5-year overall survival was significantly higher in the PS-matched resection group than in the high-risk TACE group according to the HAP score (B). PS, propensity score; TACE, trans-arterial chemoembolization; HAP, hepatoma arterial embolization prognostic score.

The patients in the TACE group were stratified according to their HAP scores. Patients were divided into low- (HAP A and B classes; *n*=535, 44.4%) and high-risk groups (HAP C and D classes; *n*=671, 55.6%). Low-risk patients in the TACE group had a significantly higher 5-year OS than those in the high-risk group (aHR=1.457, 95% CI: 1.243–1.707, *P*<0.001) (Fig. 4A). In the PS-matched cohort of the high-risk TACE and resection groups, the resection group was significantly associated with a higher 5-year OS than the TACE group (aHR=2.340, 95% CI: 1.818–3.012, *P*<0.001) (Fig. 4B).

Patients were divided into low- (mHAP-II B and C classes; *n*=535, 44.4%) and high-risk (mHAP-II D class; *n*=671, 55.6%) groups according to the mHAP-II score. In the PS-matched cohort of the high-risk TACE and resection groups, the resection group was significantly associated with a higher 5-year OS than the TACE group (aHR=2.340, 95% CI: 1.818–3.012, *P*<0.001).

### Higher 5-year overall survival in the resection group in patients with each tumor burden in PS-matched cohorts

According to various tumor burden scores, patients were divided into low tumor burden and high tumor burden groups: low tumor burden (six-and-twelve score ≤6, up-to-seven criteria ≤7, four-to-seven criteria ≤4 tumors and ≤7 cm, BCLC-B subclassification=B1, Albumin-platelet index >781.2, and Multiplication of tumor maximum diameter and number ≤12) and high tumor burden (six-and-twelve score >6, up-to-seven criteria >7, four-to-seven criteria >4 tumors or >7 cm, BCLC-B subclassification=B2 and B3, Albumin-platelet index ≤781.2, and Multiplication of tumor maximum diameter and number >12).

Among patients who had low tumor burden for each score in the PS-matched cohort, the resection group showed a significantly higher 5-year OS than the TACE group (all *P*<0.05). Finally, among patients who had high tumor burden for each score in the PS-matched cohort, the resection group showed a significantly higher 5-year OS than the TACE group (all *P* <0.05) (Table 4).

**Table 4:** Risk reduction for 5-year overall survival of resection over TACE in patients who have each tumor burden in the propensity score-matched cohort.

	Multivariable analysis		
Model	aHR	95% CI	*P*
Low tumor burden^a^			
Six-and-twelve score (*n*=349)	2.150	1.661–2.782	<0.001
Up-to-seven criteria (*n*=83) (=the Kinki criteria in the intermediate-stage)	2.061	1.170–3.632	0.012
Four-seven criteria (*n*=265)	2.891	2.053–4.072	<0.001
BCLC-B subclassification (*n*=185)	2.814	1.885–4.200	<0.001
Albumin-platelet index	2.987	2.292–3.891	<0.001
Multiplication of tumor maximum diameter and number	2.963	2.156–4.072	<0.001
High tumor burden^a^			
Six-and-twelve score (*n*=64)	4.470	2.611–7.654	<0.001
Up-to-seven criteria (*n*=215) (=the Kinki criteria in the intermediate-stage)	2.024	1.518–2.699	<0.001
Four-seven criteria (*n*=126)	1.697	1.204–2.390	0.002
BCLC-B subclassification (*n*=212)	2.056	1.536–2.754	<0.001
Albumin-platelet index	2.557	1.934–3.381	<0.001
Multiplication of tumor maximum diameter and number	2.160	1.666–2.800	<0.001

a Low tumor burden (six-and-twelve score ≤6, up-to-seven criteria ≤7, four-to-seven criteria ≤4 tumors and ≤7 cm, BCLC-B subclassification=B1, Albumin-platelet index >781.2, and Multiplication of tumor maximum diameter and number ≤12) and high tumor burden (six-and-twelve score >6, up-to-seven criteria >7, four-to-seven criteria >4 tumors or >7 cm, BCLC-B subclassification=B2 and B3, Albumin-platelet index ≤781.2, and Multiplication of tumor maximum diameter and number >12).

aHR, adjusted hazard ratio; TACE, trans-arterial chemoembolization.

## Discussion

Although the standard treatment for patients with intermediate-stage HCC is TACE^3^, it may not be appropriate for all patients, such as those with TACE refractoriness or resectable tumors. Recently, tyrosine kinase inhibitors and immune checkpoint inhibitors have been suggested as alternatives for the treatment of intermediate-stage HCC^6,24^, particularly in patients with poor prognosis due to TACE refractoriness. Although resection can be considered for the treatment of intermediate-stage HCC, clinical decisions differ among centers because it is unclear who can have clinical benefits from resection over TACE in patients with intermediate-stage HCC^8,25,26^.

This study has several important clinical implications and strengths. First, among patients with intermediate-stage HCC, we found that resection was significantly associated with better clinical outcomes than TACE in the PS-matched cohort: 47.7% (vs. 19.8%) for 2-year RFS and 67.0% (vs. 31.7%) for 5-year overall survival. This result is in line with previous studies suggesting the clinical benefits of resection over TACE in intermediate-stage HCC^8,23,24^. A previous meta-analysis of 2619 patients with intermediate-stage HCC demonstrated that resection resulted in better overall survival than TACE, without any significant increase in postoperative complications or 30-day mortality rates^27^.

Second, we developed a new score that enables risk assessment for 2-year RFS, i.e., early recurrence in patients with intermediate-stage HCC who undergo resection, using baseline liver function (ALBI grades) and tumor burden factors such as serum AFP levels, tumor number, and tumor size. While multivariate statistical analysis involved a limited number of covariates, the use of variables sourced from the extensive cancer registration database, KPLCR, offers distinct advantages including enhanced universality. The score showed excellent predictive performance (AUC ≥0.80) for 2-year RFS in both the training and validation cohorts treated with resection. Commonly measured parameters, and the simple calculation of the score could be implemented for routine clinical use. Although it has been known that early recurrence mainly occurs due to high tumor burden, liver function (ALBI grades) could also affect the 2-year RFS in this study, and it was similar to results of a previous study using the ALBI grade^20^. The underlying reason for the association between liver dysfunction and early recurrence remains unclear. However, attenuation of immunity for HCC control according to liver dysfunction may be one of the reasons^28^. Based on the new score, patients treated with resection can be divided into two groups: low-risk (<4.24, 46.5%) and high-risk (≥4.24, 53.5%). In the low-risk group, the resection showed a significant association with a higher 2-year RFS than TACE did after PS-matching analyses; however, in the high-risk group, there was no significant difference. Given that the benefits from resection and TACE differ according to individual risk, it may be important to assess the risk of early recurrence in patients treated with resection.

Third, among patients treated with TACE who were predicted to have a poor prognosis on TACE risk scores, indicating a high possibility of TACE refractoriness, resection showed a significant association with a higher 5-year overall survival than TACE did. The SPACE trial compared the RFS between TACE alone and TACE with sorafenib in patients with intermediate-stage HCC; the RFS failed to improve^29^. In contrast, another clinical trial (the TACTICS trial)^30^ showed a significantly better progression-free survival in patients treated with TACE and sorafenib than TACE alone, showing that the risk reduction for progression-free survival in patients treated with TACE and sorafenib was 41.0%. Given that the overall improvement in RFS in the resection group was 47.1% compared to the TACE group after PS-matching analyses in this study, our findings suggest that resection may be a better alternative treatment for patients with intermediate-stage HCC who were predicted to have a poor prognosis.

Furthermore, resection showed a higher 5-year overall survival than TACE did even in patients with a high tumor burden after PS-matching analyses. Microvascular invasion is a well-known independent prognostic factor^31^. As the tumor burden within the intermediate-stage HCC increases, the possibility of microvascular invasion increases, thereby affecting RFS and OS^32^. In our study, microvascular invasion was a significant risk factor for 2-year RFS (aHR=1.112, 95% CI: 1.004–1.201, *P*<0.001) among patients treated with resection. Compared with TACE, curative resection can reduce the possibility of remnant HCC cells invading the microvessels and progressing to intrahepatic metastasis. In addition, previous studies have shown immune restoration after successful removal of the tumor and tumor-related microenvironment and the association of immune recovery and decreased HCC recurrence^28^. The restoration of the immune system after resection might increase RFS and OS in patients with intermediate-stage HCC compared to those after TACE.

To determine the first-line treatment for intermediate-stage HCC, OS may not be a suitable primary endpoint because high rates of early recurrence which account for greater than 70% of tumor recurrence^33^. Identification of patients who are at high-risk of recurrence after curative resection allows clinicians to provide appropriate surveillance to detect recurrent HCC at its earliest when curative therapy may still be feasible.

This study had several limitations. First, because of its retrospective nature, selection bias cannot be completely avoided, even if we used PS-matching analyses to eliminate the inherent differences between the two groups. It may be hypothesized that patients with preserved liver function and good functional status are preferentially selected as surgical candidates. However, a randomized controlled trial and meta-analysis regarding this issue have already demonstrated similar results to those found in this study^34^. Second, this study mainly included patient cohorts in which hepatitis B virus was the predominant etiology of HCC, which is quite different from that in Western countries. Given that HCC prognosis can differ according to etiologies, it is necessary to validate the predictive performance of the SR-B score in intermediate-stage HCC due to other etiologies. Finally, data regarding doses of chemotherapeutic agents and frequency of TACE were not collected. As a result, these critical risk factors for recurrence were not assessed in our study.

In conclusion, our score composed of ALBI scores, serum AFP levels, tumor number, and tumor size can predict early recurrence after resection in patients with intermediate-stage HCC. This score and risk stratification can be a useful tool to guide appropriate strategy for patients who may benefit from resection and require stricter surveillance. Further prospective studies are required to explore the clinical applicability of this score in patients with frequent follow-up and clinical trials of adjuvant therapy.

## Ethical approval

The study protocol was in accordance with the ethical guidelines of the 1975 Declaration of Helsinki and approved by the Institutional Review Board of Ewha Womans University Medical Center (IRB Number 2021-04-008, 2022AN0150).

## Consent

The requirement for written informed consent was waived by Institutional Review Board of Ewha Womans University Mokdong Hospital owing to the retrospective nature of the study.

## Sources of funding

This study was supported by the Basic Science Research Program through the National Research Foundation of Korea (NRF) funded by the Ministry of Education (Grant No: 2017R1D1A1B03031499, 2022R1I1A1A01065244, and 2022R1I1A1A01068809), the NRF funded by the Korean government (Ministry of Science and ICT) (Grant No: 2020R1C1C1004112). The funders had no role in the study design, data collection and analysis, decision to publish, or manuscript preparation.

## Author contribution

Authors make substantial contributions to conception and design, and/or acquisition of data, and/or analysis and interpretation of data: H.A.L., J.-J.Y., H.S.C., Y.P., H.Y.K., T.H.K., Y.S.S., D.H.S., and M.L.; Authors participate in drafting the article or revising it critically for important intellectual content: H.A.L., J.-J.Y., and M.L.; Authors give final approval of the version to be published: H.A.L., J.-J.Y., H.S.C., Y.P., H.Y.K., T.H.K., Y.S.S., D.H.S., and M.L.

## Conflicts of interest disclosure

The authors declare that they have no financial conflict of interest with regard to the content of this report.

## Research registration unique identifying number (UIN)

1. Name of the registry: not applicable.
2. Unique identifying number or registration ID: not applicable.
3. Hyperlink to your specific registration (must be publicly accessible and will be checked): not applicable.

## Guarantor

Minjong Lee.

## Data availability statement

Data, analytical methods, and study materials will be made available to other researchers from the corresponding author upon reasonable request.

## Provenance and peer review

Not commissioned, externally peer-reviewed.

## Supplementary Material
