# Supplementary material for: Identification of patients with favorable prognosis after resection in intermediate-stage-hepatocellular carcinoma
Source: Int J Surg. 2023 Nov 24;110(2):1008–18. doi: 10.1097/JS9.0000000000000941 (PMC10871631; doi:10.1097/JS9.0000000000000941)

**SUPPLEMENTARY MATERIAL**

**Identification of patients with favorable prognosis after resection in intermediate-stage hepatocellular carcinoma**

Han Ah Lee^1,2,4^, Minjong Lee^1,2,4†^, Jeong-Ju Yoo^3^, Ho Soo Chun^1,4^, Yewan Park^5^, Hwi Young Kim^1,4^, Tae Hun Kim^1,4^, Yeon Seok Seo^6^, and Dong Hyun Sinn^7†^

^1^Department of Internal Medicine, Ewha Womans University College of Medicine, Seoul, Korea

^2^The Korean Liver Cancer Association, Seoul, Korea

^3^Department of Internal Medicine, Soonchunhyang University Bucheon Hospital, Bucheon, Korea

^4^Department of Internal Medicine, Ewha Womans University Medical Center, Seoul, Korea

^5^Department of Internal Medicine, Kyung Hee University Hospital, Seoul, Korea

^6^Department of Internal Medicine, Korea University College of Medicine, Seoul, Korea

^7^Department of Medicine, Samsung Medical Center, Sungkyunkwan University School of Medicine, Seoul, Korea

^†^**Co-correspondence**

Minjong Lee, M.D., Ph.D.

Department of Internal Medicine, Ewha Womans University College of Medicine

260 Gonghang-daero, Gangseo-gu, Seoul, 07804, Republic of Korea

Tel: +82-2-6986-1761

E-mail: minjonglee2@naver.com or minjonglee2@ewha.ac.kr

Dong Hyun Sinn, M.D., Ph.D.

Department of Medicine, Samsung Medical Center,

Sungkyunkwan University School of Medicine, Seoul, Korea

81 Irwon-ro, Gangnam-gu, Seoul 06351, Korea

Tel: +82-2-3410-3409, Fax: +82-2-3410-6983; E-mail: dh.sinn@samsung.com

**Table of Contents**

[Supplementary Document. Study population 3](#_Toc142843395)

[Supplementary Table 1. Summary of various prediction models 4](#_Toc142843396)

[Supplementary Table 2. Diagnostic accuracy of various models for 2-year recurrence-free survival in the validation cohort 6](#_Toc142843397)

[Supplementary Table 3. Clinical characteristics of low-risk and high-risk groups according to the SR-B score](#_Toc142843398) 7

[Supplementary Figure 1. Patient flowchart](#_Toc142843399) 8

[Supplementary Figure 2. Area under the curve analysis of the SR-B score](#_Toc142843400) 9

[Supplementary Figure 3. The calibration plots of the SR-B score for 2-year recurrence-free survival](#_Toc142843401) 9

[Supplementary Figure 4. The 2-year recurrence-free survival of the resection and TACE groups after PS matching 1](#_Toc142843402)0

[Supplementary Figure 5. The 5-year overall survival of the resection and TACE groups after PS matching 1](#_Toc142843403)0

**Supplementary Document.** Study population

1. Korean Primary Liver Cancer Registry (KPLCR) (n=815)
2. Soonchunhyang University Bucheon Hospital, Bucheon-si, Gyeonggi-do, Korea (n=20)
3. Ewha Womans University Seoul Hospital, Seoul, Korea (n=51)
4. Ewha Womans University Mokdong Hospital, Seoul, Korea (n=48)
5. Korea University Anam Hospital, Seoul, Korea (n=76)
6. Korea University Ansan Hospital, Ansan, Korea (n=17)
7. Samsung Medical Center, Seoul, Korea (n=659)

**Supplementary Table 1.** Summary of various prediction models

| Author | Model name | Variables and risk allocation | Scoring | |
| --- | --- | --- | --- | --- |
| Kadalayil et al. (15) | HAP | Serum albumin < 3.6 g/dL | 1 point | |
|  |  | AFP > 400 ng/mL | 1 point | |
|  |  | Total bilirubin > 17 μmol/L | 1 point | |
|  |  | Tumor size > 7 cm | 1 point | |
|  |  | HAP A class | 0 point | |
|  |  | HAP B class | 1 point | |
|  |  | HAP C class | 2 points | |
|  |  | HAP D class | >2 points | |
| Cappelli et al. (16) | Modified HAP-II | Tumor size > 7 cm | 1 point | |
|  |  | Tumor numbers ≥ 2 | 1 point | |
|  |  | AFP > 400 ng/mL | 1 point | |
|  |  | Total bilirubin > 0.9 mg/dL | 1 point | |
|  |  | Serum albumin < 3.6 g/dL | 1 point | |
|  |  | mHAP-II A class | 0 point | |
|  |  | mHAP-II B class | 1 point | |
|  |  | mHAP-II C class | 2 points | |
|  |  | mHAP-II D class | 3-5 points | |
| Mazzaferro et al. (17) | Up-to-seven | Largest tumor size (cm) + number of tumors | | |
|  |  | ≤ 7 | In | |
|  |  | > 7 | Out | |
| Wang et al. (18) | Six-and-twelve | Largest tumor size (cm) + number of tumors | | |
|  |  | ≤ 6 | Low risk | |
|  |  | < 6, but ≤ 12 | Moderate risk | |
|  |  | > 12 | High risk | |
| Yamakado et al. (19) | Four-and-seven criteria | Number of tumors and largest tumor size (cm) | | |
|  |  | ≤ 4 tumors and ≤ 7 cm | In | |
|  |  | > 4 tumors or > 7 cm | Out | |
| Bolondi et al. (4) | BCLC-B subclassification | CTP score 5-7  Beyond Milan and within up-to-seven | B1 | |
|  |  | CTP score 5-6  Beyond up-to-seven | B2 | |
|  |  | CTP score 7  Beyond up-to-seven | B4 | |
|  |  | CTP score 8-9  Any tumors | B4 | |
| Chan et al. (20) | ERASL-pre score | 0.818 × Gender (0: Female, 1: Male) + 0.447 × Albumin-Bilirubin grade (0: Grade 1; 1: Grade 2 or 3) + 0.100 × ln(Serum AFP in lg/L) + 0.580 × ln(Tumor size in cm) + 0.492 × Tumor number (0: Single; 1: Two or three; 2: Four or more) | | |
|  |  | ≤ 2.558 | | Low risk |
|  |  | > 2.558 to ≤ 3.521 | | Intermediate risk |
|  |  | > 3.521 | | High risk |
| Kudo et al. (21) | Kinki Criteria | CTP score 5-7  Beyond Milan and within up-to-seven | B1 | |
|  |  | CTP score 5-7  Beyond up-to-seven | B2 | |
|  |  | CTP score 8,9  Any tumors | B3 | |
| Yugawa et al. (9) | Albumin-platelet index | 156.2 × albumin (g/dL) + platelet count (×10^9^/L) | | |
|  |  | >781.2 | B1 | |
|  |  | ≤781.2 | B2 | |
| Morine et al. (10) | Multiplication of tumor maximum diameter and number | Tumor maximum diameter × number | | |
|  |  | >12 | High risk | |
|  |  | ≤12 | Low risk | |

HAP, hepatoma arterial embolization prognostic score; AFP, alpha-fetoprotein; ERASL, Early Recurrence After Surgery for Liver tumor; BCLC, Barcelona Clinic Liver Cancer; CTP, Child-Turcotte-Pugh

**Supplementary Table 2.** Diagnostic accuracy of various models for 2-year recurrence-free survival in the validation cohort

| Model | AUC value | 95% Confidence Interval | *P* value |
| --- | --- | --- | --- |
| SR-B score | 0.801 | 0.733–0.864 | reference |
| Serum alpha-fetoprotein | 0.712 | 0.635–0.790 | <0.001 |
| ERASL-pre score | 0.715 | 0.638–0.793 | <0.001 |
| Up-to-seven (=Kinki criteria) | 0.666 | 0.595–0.737 | <0.001 |
| BCLC-B subclassification | 0.661 | 0.588–0.733 | <0.001 |
| HAP score | 0.658 | 0.581–0.734 | <0.001 |
| mHAP-II score | 0.658 | 0.581–0.734 | <0.001 |
| Four-and-seven criteria | 0.636 | 0.565–0.706 | <0.001 |
| Six-and-Twelve score | 0.600 | 0.529–0.671 | <0.001 |
| Albumin-platelet index | 0.490 | 0.438-0.542 | <0.001 |
| Multiplication of tumor maximum diameter and number | 0.648 | 0.599-0.698 | <0.001 |

AUC, area under the curve; SR-B score, Surgery risk score in BCLC-B; HAP, hepatoma arterial embolization prognostic; BCLC, Barcelona Clinic Liver Cancer, ERASL, Early Recurrence After Surgery for Liver tumor

**Supplementary Table 3.** Clinical characteristics of low- risk and high-risk groups according to the SR-B score

|  | Low-risk group  (n=223, 46.5%) | High-risk group  (n=257, 53.5%) | *P* value |
| --- | --- | --- | --- |
| Age | 61.0 (55.0–68.0) | 58.0 (52.0–69.0) | 0.056 |
| Male, n (%) | 190 (85.2) | 211 (82.1) | 0.361 |
| Diabetes, n (%) | 52 (23.3) | 56 (21.8) | 0.689 |
| Viral etiology, n (%) | 157 (70.4) | 194 (75.5) | 0.210 |
| Serum albumin, g/dL | 4.3 (4.0–4.5) | 4.0 (3.7–4.3) | <0.001 |
| Total bilirubin, mg/dL | 0.7 (0.5–0.9) | 0.7 (0.4–1.0) | 0.459 |
| INR | 1.03 (0.99–1.08) | 1.04 (1.00–1.12) | 0.103 |
| Alanine aminotransferase, IU/L | 33.0 (22.0–51.0) | 39.0 (25.5–59.0) | 0.680 |
| Platelet count, × 10^9^/L | 185.0 (138.0–219.0) | 185.0 (143.0–237.5) | 0.423 |
| Alpha-fetoprotein, ng/mL | 4.8 (3.0–76.0) | 67.7 (9.5–1020.8) | <0.001 |
| ALBI grade, n (%) |  |  | <0.001 |
| 1 | 192 (86.1) | 153 (59.5) |  |
| 2 | 30 (13.5) | 96 (37.4) |  |
| 3 | 1 (0.4) | 8 (3.1) |  |
| MELD score | 7.0 (6.5–8.0) | 7.0 (6.4–8.0) | 0.873 |
| Child-Pugh class, n (%) |  |  | 0.004 |
| A | 219 (98.2) | 238 (92.6) |  |
| B | 4 (1.8) | 19 (7.4) |  |
| Tumor number ≥ 3, n (%) | 18 (8.1) | 88 (34.2) | <0.001 |
| Maximal tumor size ≥ 5 cm, n (%) | 38 (17.0) | 179 (69.6) | <0.001 |

Variables are expressed as median (interquartile range) or n (%). SR-B score, Surgery risk score in BCLC-B; INR, international normalized ratio; ALBI, albumin-bilirubin; MELD, model for end-stage liver disease

**Supplementary Figure 1.** Patient flowchart

Abbreviations: HCC, hepatocellular carcinoma; TACE, trans-arterial chemoembolization


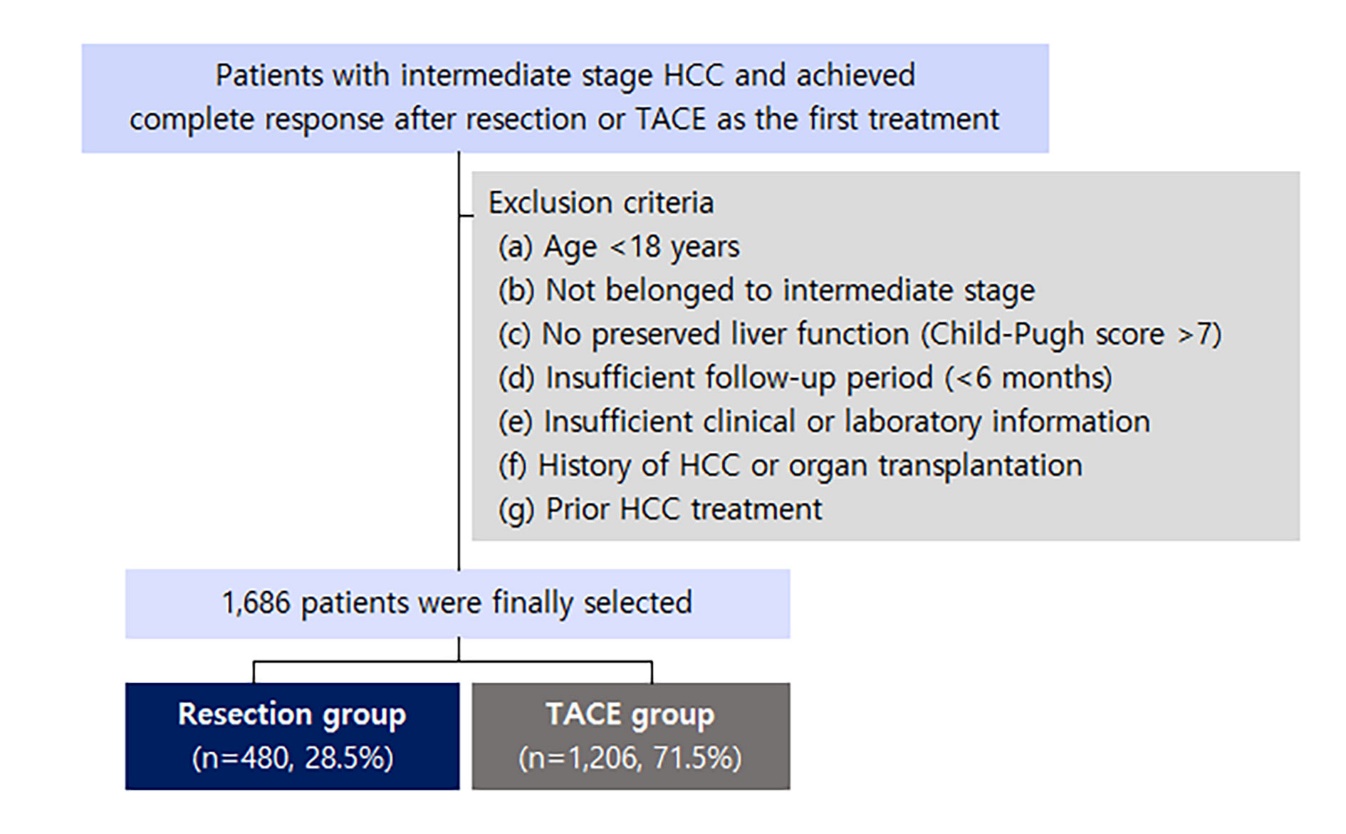


**Supplementary Figure 2.** Area under the curve analysis of the SR-B score in the training (A), validation (B), and entire cohorts (C).

Abbreviations: SR-B score, surgery risk score in BCLC-B


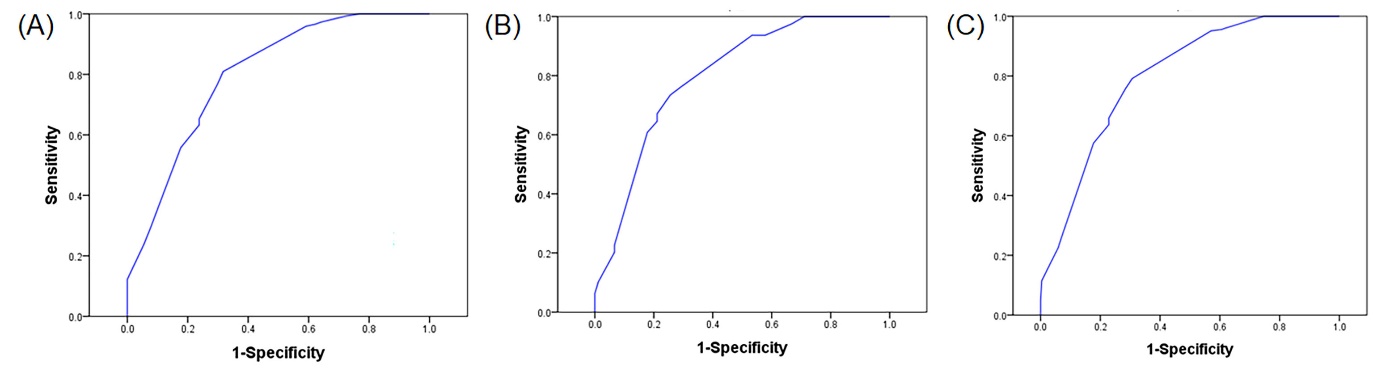


**Supplementary Figure 3.** The calibration plots of the SR-B score for 2-year recurrence-free survival in the training (A) and validation (B) cohorts.

Abbreviations: SR-B score, surgery risk score in BCLC-B


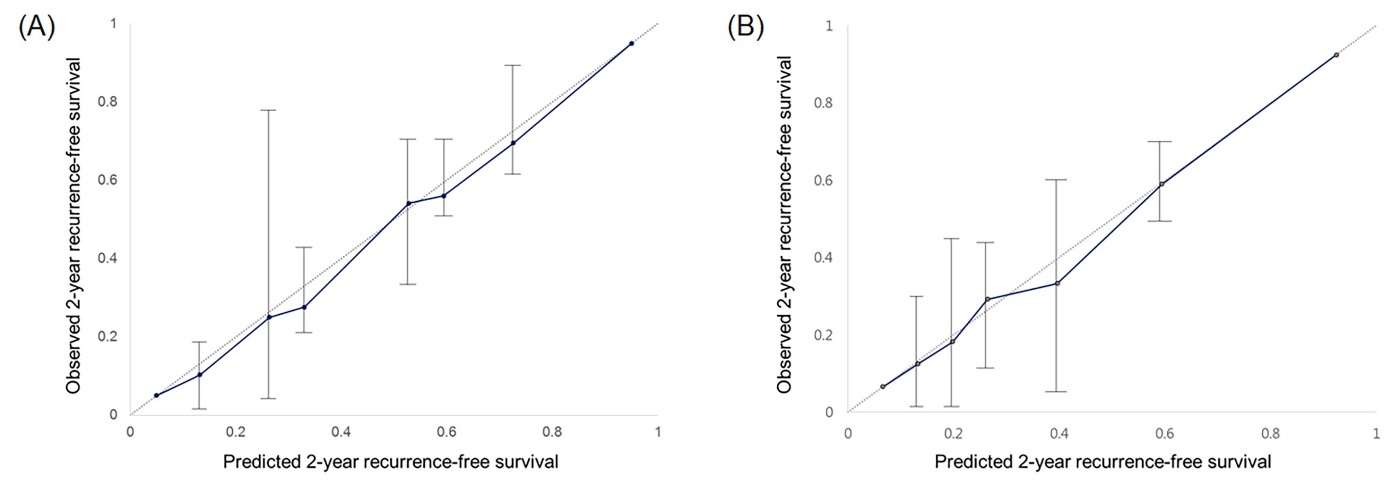


**Supplementary Figure 4**. The 2-year recurrence-free survival of the resection and TACE groups after PS matching. The 2-year recurrence-free survival was significantly higher in the resection group at low risk according to the SR-B score than in the TACE group after PS matching (A). The 2-year recurrence-free survival was comparable between the resection group at high risk according to the SR-B score and the TACE group after PS matching (B).

Abbreviations: SR-B score, surgery risk score in BCLC-B; PS, propensity score; TACE, trans-arterial chemoembolization.

**
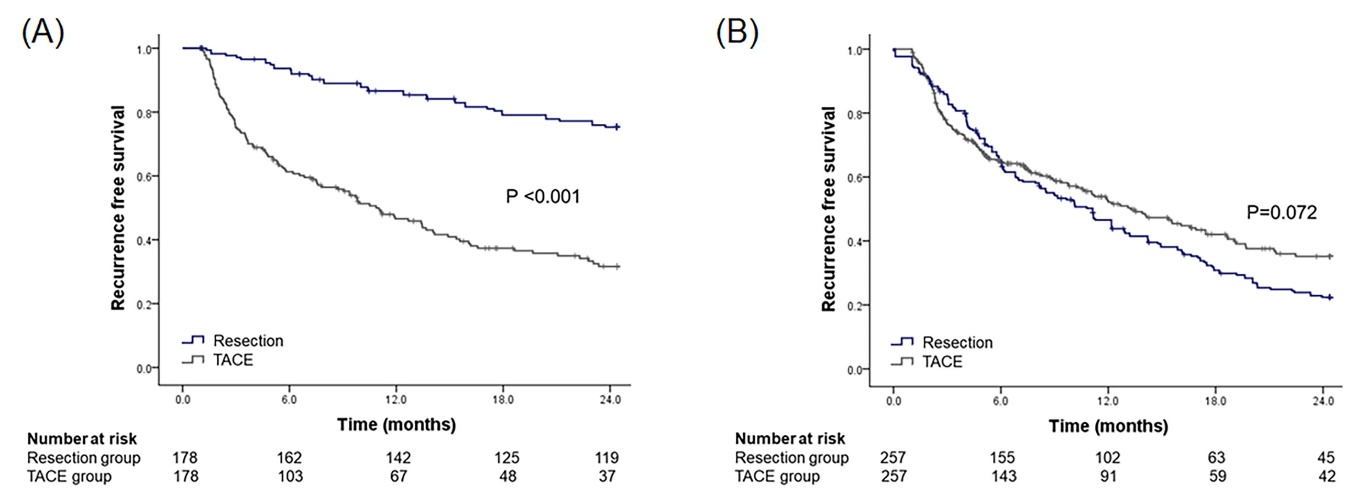
**

**Supplementary Figure 5**. The 5-year overall survival of the resection and TACE groups after PS matching. The 5-year overall survival was significantly higher in the resection group at low risk according to the SR-B score than in the TACE group after PS matching (A). The 5-year overall survival was significantly higher in the resection group at high risk according to the SR-B score than in the TACE group after PS matching (B).

Abbreviations: SR-B score, surgery risk score in BCLC-B; PS, propensity score; TACE, trans-arterial chemoembolization.


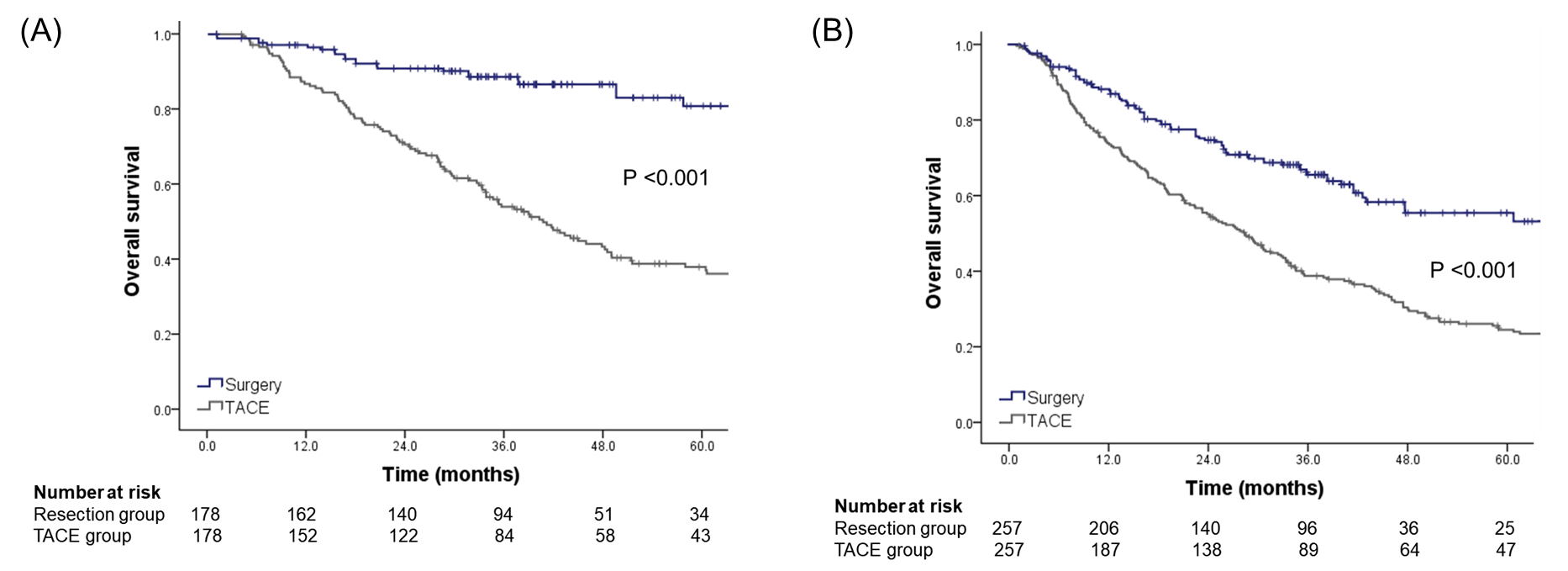

Supplement: Supplementary file 1 [file js9-110-1008-s001.docx]
